# APOE genotype-dependent differences in human astrocytic energy metabolism

**DOI:** 10.3389/fncel.2025.1603657

**Published:** 2025-09-01

**Authors:** Vanessa Budny, Chantal Bodenmann, Kathrin J. Zürcher, Maik Krüger, Sherida M. de Leeuw, Rebecca Z. Weber, Ruslan Rust, Luca Ravotto, Iván Ruminot, L. Felipe Barros, Bruno Weber, Christian Tackenberg

**Affiliations:** 1 Institute for Regenerative Medicine, University of Zurich, Schlieren, Switzerland; 2 Neuroscience Center Zurich, University of Zurich and ETH, Zurich, Switzerland; 3 Department of Physiology and Neuroscience, University of Southern California, Los Angeles, CA, United States; 4 Zilkha Neurogenetic Institute, Keck School of Medicine, University of Southern California, Los Angeles, CA, United States; 5 Institute for Pharmacology and Toxicology, University of Zurich, Zürich, Switzerland; 6 Centro de Estudios Científicos (CECs), Valdivia, Chile; 7 Facultad de Ciencias para el Cuidado de la Salud, Universidad San Sebastián, Valdivia, Chile; 8 Facultad de Medicina, Universidad San Sebastián, Valdivia, Chile

**Keywords:** apolipoprotein E (APOE), Alzheimer’s disease (AD), induced pluripotent stem cells (iPSCs), human astrocytes, energy metabolism, glycolysis, mitochondrial function, mitochondrial uncoupling

## Abstract

**Introduction:**

The main genetic risk factor for Alzheimer’s disease (AD) is the presence of the apolipoprotein E4 (*APOE4*) allele. While *APOE4* increases the risk of developing AD, the *APOE2* allele is protective and *APOE3* is risk-neutral. In the brain, APOE is primarily expressed by astrocytes and plays a key role in various processes including cholesterol and lipid transport, neuronal growth, synaptic plasticity, immune response and energy metabolism. Disruptions in brain energy metabolism are considered a major contributor to AD pathophysiology, raising a key question about how different APOE isoforms affect the energy metabolism of human astrocytes.

**Methods:**

In this study, we generated astrocytes (iAstrocytes) from *APOE*-isogenic human induced pluripotent stem cells (iPSCs), expressing either APOE2, APOE3, APOE4 or carrying an APOE knockout (*APOE-KO*), and investigated *APOE* genotype-dependent changes in energy metabolism.

**Results:**

ATP Seahorse assay revealed a reduced mitochondrial and glycolytic ATP production in *APOE4* iAstrocytes. In contrast, glycolysis stress tests demonstrated enhanced glycolysis and glycolytic capacity in *APOE4* iAstrocytes while genetically encoded nanosensor-based FLIM analysis revealed that *APOE* does not affect lactate dynamics. In agreement with the increased glycolytic activity, *APOE4* iAstrocytes also showed elevated mitochondrial respiration and activity, indicated by proteomic GO enrichment analysis and mitochondrial stress test. This was accompanied by elevated proton leak in *APOE4* iAstrocytes while levels of mitochondrial uncoupling proteins (UCPs) were not affected. Mass spectrometry-based metabolomic analysis identified various energy and glucose metabolism-related pathways that were differentially regulated in *APOE4* compared to the other genotypes, including mitochondrial electron transport chain (ETC) and glycolysis. In general, *APOE2* and *APOE-KO* iAstrocytes showed a very similar phenotype in all functional assays and differences between *APOE2*/*APOE-KO* and *APOE4* were stronger than between *APOE3* and *APOE4*.

**Discussion:**

Our study provides evidence for *APOE* genotype-dependent effects on astrocyte energy metabolism and highlights alterations in the bioenergetic processes of the brain as important pathomechanisms in AD.

## 1 Introduction

The human brain is a highly energy-demanding organ and requires substantial metabolic resources to maintain its functions. Alterations in the bioenergetic processes of the brain are commonly observed during aging and are implicated in various neurodegenerative diseases, such as Alzheimer’s disease (AD). AD is the most common age-related neurodegenerative disease affecting memory and executive functions. Several genetic and non-genetic risk factors have been identified so far to influence the AD risk. Among those, the ε4 allele of the apolipoprotein E (*APOE4*) represents the strongest genetic risk factor (Tanzi, 2012). Three major allelic variants of human *APOE* have been identified: *APOE2*, *APOE3* and *APOE4*. *APOE4* increases the risk for developing AD by 2.5-4-fold in heterozygous and 12-15-fold in homozygous carriers (Corder et al., 1993; Grupe et al., 2007; Corneveaux et al., 2010), however the risk is affected by factors such as sex or population ancestry (Belloy et al., 2023). In contrast, *APOE3* is defined as risk-neutral while *APOE2* represents the strongest genetic protective factor (Corder et al., 1994; Farrer et al., 1997; Reiman et al., 2020). In the brain, APOE is mainly produced by astrocytes, which generate up to 80% of total brain APOE (Blumenfeld et al., 2024). APOE isoforms differentially affect astrocyte function (de Leeuw and Tackenberg, 2019). Human isogenic iPSC-derived astrocytes expressing APOE4 displayed a pro-inflammatory phenotype, reduced beta-amyloid uptake capacity and altered lipid metabolism compared to astrocytes expressing APOE3 or APOE2 (de Leeuw et al., 2022). Importantly, *APOE* is also implicated in regulating bioenergetic homeostasis of the brain.

Numerous studies have demonstrated altered glucose metabolism as a common phenotype in AD, especially in *APOE4* carriers. [^18^F] fluorodeoxyglucose (FDG)-PET imaging showed hypometabolism in AD brains, mainly in the temporoparietal cortex including the precuneus and posterior cingulate cortex (Reiman et al., 1996; Foster et al., 2007). Interestingly, several studies in young *APOE4* carriers or in *APOE4*-knock-in mice with no apparent neurodegenerative pathology, revealed increased brain metabolism and activity (Filippini et al., 2009; Evans et al., 2017; Nuriel et al., 2017; Venzi et al., 2017), indicating that the *APOE4* effect on brain energy metabolism may differ depending on the degree of pathology. Further, post-mortem analysis of AD brains discovered higher brain tissue glucose concentration, reduced glycolytic flux and lower levels of neuronal glucose transporter GLUT3 but no effect on astrocytic glucose transporter GLUT1, suggesting cell type specific metabolic alterations in AD (An et al., 2018).

The proposed mechanisms of how *APOE* variants, especially *APOE4*, affect the energy metabolism of neural cells remain inconclusive. They range from reduced glycolysis in APOE4-expressing cells (Wu et al., 2018; Fang et al., 2021; Zhang et al., 2023), via a metabolic shift from oxidative phosphorylation to glycolysis in *APOE4* or AD patient cells (Sonntag et al., 2017; Williams et al., 2020; Farmer et al., 2021; Lee et al., 2023) to an increase in oxidative phosphorylation in *APOE4* induced neurons (Budny et al., 2024). Further, many studies only compare two *APOE* variants, mostly *APOE4* and *APOE3*. Thus, more research is required to specifically show how *APOE4*, compared to both *APOE3* and *APOE2*, alters glycolytic and mitochondrial function in human brain cells.

In the present study, we used human *APOE*-isogenic iPSC-derived astrocytes (iAstrocytes) to investigate the impact of *APOE4*, *APOE3*, *APOE2* and *APOE-KO* on astrocyte energy metabolism. Our findings reveal *APOE* genotype-dependent metabolic and proteomic changes. *APOE4* iAstrocytes showed upregulated oxidative phosphorylation (OXPHOS) proteomic pathways, accompanied by increased mitochondrial respiration and glycolysis. Interestingly, despite the elevated metabolic activity, *APOE4* iAstrocytes produced less ATP compared to the other *APOE* variants. The substantial differences observed in comparison to *APOE-KO* indicate a gain-of-function mechanism by *APOE4* rather than a loss-of-function.

## 2 Materials and methods

### 2.1 iPS cell culture

*APOE*-isogenic iPS cell lines BIONi10-C3 (*APOE-KO*), BIONi10-C6 (*APOE2*), BIONi10-C2 (*APOE3*), and BIONi10-C4 (*APOE4*); (Schmid et al., 2019; Schmid et al., 2020; de Leeuw et al., 2022) have been purchased from EBiSC. iPSCs were cultured on vitronectin (1:25; 100–0763, StemCell Technologies) coated plates in mTESR+ medium (100–0276, StemCell Technologies), split every 3–4 days and culture medium was exchanged every other day. For splitting, cells were washed with DPBS, incubated with ReLeSR (5,872, StemCell Technologies) for 4 min at 37°C with 5% CO2, detached in 1 mL mTESR+ and transferred to a new plate.

### 2.2 iAstrocyte differentiation

iAstrocyte differentiation was carried out as described earlier (de Leeuw et al., 2022). In brief, iPSC lines were differentiated into NPCs by applying dual SMAD inhibition. On day 0, cells (appr. 20% confluent) were washed with PBS and medium changed to Neural Induction Medium 1, including 50% advanced DMEM/F12 (21,331,020, Gibco), 50% Neurobasal (21103–049, Gibco), 1x N2 (17,502,048, Thermo scientific), 1x B27 (17,504,044, Thermo Scientific), 2 mM Glutamax (Gibco) and 10 ng/mL hLIF (AF-300-05, Peprotech), 4 μM CHIR99021 (130–103-926, Miltenyi), 3 μM SB431542 (130–106-543, Miltenyi), 2 μM Dorsomorphin (130–104-466, Miltenyi), and 0.1 μM Compound E (73,952, StemCell Technologies). Medium was changed daily. On day 3, cells were washed with PBS and medium was changed to Neural Induction Medium 2, including 50% advanced DMEM/F12, 50% Neurobasal, 1x N2, 1x B27, 2 mM Glutamax, 10 ng/mL hLIF, 4 mM CHIR99021, 3 mM SB431542, and 0.1 mM Compound E. On day 7, cells (appr. 80% confluent) were passaged from 12 well plates to 6 well plates coated with 15 mg/mL poly-L-ornithine (P4957, Sigma-Aldrich) and 10 mg/mL laminin (L2020, Sigma-Aldrich). The medium was changed to neural stem cell maintenance medium (NSMM), including 50% advanced DMEM/ F12, 50% Neurobasal, 1x N2, 1x B27, 2 mM Glutamax, 10 ng/mL hLIF, 3 μM CHIR99021, and 2 μM SB431542. Medium was changed every day and cells were passaged twice a week (at 90–100% confluency). The first five passages 2 μM Thiazovivin (SML1045, Sigma) was added to NSMM and after five passages 5 ng/mL FGF (PHG6015, Thermo Fisher) and 5 ng/mL epidermal growth factor (EGF; PHG6045, Thermo Fisher) was added.

NPC passages 1–4 were further differentiated to astrocytes replating them onto 1 mg/mL Fibronectin (F1141-1MG, Sigma-Aldrich; 135.000 cells/well in 6 well plates) coated plates and changing the medium 1 day later to complete astrocyte medium (AM; 1801, ScienCell) supplemented with 2% FCS (0010, ScienCell) and AGS (1852, ScienCell) and P/S (0503, ScienCell) for 30 days. Medium was changed every other day and cells were passaged at 80–100% confluency. For the main stock, cells were frozen in BAMBANKER serum-free cryopreservation medium (WAKO302-14681, Avantor) at d28 which was the last time point before maturation of the astrocytes. For maturation and elimination of remaining proliferative cells, astrocytes were replated (350.000 cells/well in 6 well plates) and medium was changed to AM with 2 mM AraC (C1768, Sigma) but without FCS and then changed every other day. From day 37, cells were cultured in AM without FCS and without AraC until day 45. Cells were always replated at least 24 h before an experiment.

### 2.3 Immunocytochemistry

iAstrocytes were fixated after differentiation at d45 for 20 min at room temperature (RT) with 4% Paraformaldehyde (PFA; 47377.9 L, VWR) and 4% sucrose (S9378, Merck) in DPBS. After washing the cells three times with PBS for about 5 min at RT, cells were blocked with 10% donkey serum (D9663, Sigma Aldrich) and 0.1% Triton (X100, Sigma Aldrich) in DPBS for 1 h at RT. After washing with DPBS, cells were incubated with primary antibodies diluted in 3% donkey serum and 0.1% Triton in DPBS overnight at 4°C. After washing at the next day, secondary antibodies diluted in 3% donkey serum and 0.1% Triton in DPBS were added to the cells and incubated for 2 h at RT in the dark. After washing, cells were incubated with 0.4 ng/μl DAPI (D9542, Sigma Aldrich) diluted in DPBS for 10 min. After another washing step, the coverslips were mounted with Mowiol (81,381, Sigma Aldrich) on microscope objectives and stored over night at 4°C protected from light.

For calculation of differentiation efficiency, the number of s100β-positive cells was calculated per total DAPI count.

**Table 1:** 

Primary antibody	Producer	Cat. No.	Dilution
Anti-S100β	Sigma	S2532	1:200
Anti-GJA1	Abcam	Ab235585	1:200
Secondary antibody	Producer	Cat. No.	Dilution
Dk-α-ms-cy3	Jackson ImmunoResearch	715–165-151	1:1000
Dk-α-rb-cy5	Jackson ImmunoResearch	715–175-152	1:1000

### 2.4 Protein extraction

iAstrocytes were harvested with Accutase. Cells were resuspended in RIPA buffer supplemented with protease inhibitor (11,697,498,001, Sigma Aldrich). Three cycles of 30s of sonication were used to disrupt the cellular membranes. To extract the proteins, samples were centrifuged at 20.000 g for 10 min at 4°C and the supernatant was finally collected and stored at −20°C until usage. Protein concentrations were determined with the Pierce BCA Assay Kit (23,252, Thermo Fisher Scientific) according to the manufacturer’s instructions and the absorption at 562 nm was measured with the Infinite M Nano plate reader (Tecan).

### 2.5 Immunoblotting

Each sample was diluted in RIPA buffer to obtain a concentration of 10 μg protein and mixed with sample buffer (NP0007, Thermo Fisher Scientific). Samples were denatured for 5 min at 95°C. Seeblue2 plus protein ladder (LC5925, Thermo Fisher Scientific) and samples were loaded onto 10–20% Tricine SDS-PAGE gels (EC6625BOX, Invitrogen) and run at 60 V for 15 min and 100 V for 90 min. Blotting was performed with the Trans-Blot Turbo Mini 0.2 μm nitrocellulose Transfer Pack (1,704,158, Bio-Rad) and the Trans-Blot Turbo Transfer System (1,704,158, Bio-Rad) at 2.5 A with 25 V for 7 min. Membranes were washed with 0.05% Tween (P1379, Sigma Aldrich) in PBS and blocked with 5% milk solution (A0830, ITW Reagents) in PBS for 1 h at RT shaking. Membranes were then washed three times with PBS-Tween and incubated with primary antibodies diluted in 5% milk solution in PBS-Tween overnight at 4°C shaking. The next day, membranes were washed three times with PBS-Tween and incubated with secondary antibodies diluted in 5% milk solution in PBS-Tween for 2 h at RT in the dark shaking. Membranes were washed three times, developed with one of the ECL selection kits (RPN2232/ RPN2235, Cytiva; 32,106, Thermo Fisher Scientific) and imaged at the Image Quant 800 (Cytiva). Background subtraction was performed, and protein expressions were normalized to the housekeeping marker GAPDH or *α*-tubulin.

**Table 2:** 

Primary antibody	Producer	Cat. No.	Dilution
Anti-MFN1	Cell Signaling	14739S	1:250
Anti-MFN2	Cell Signaling	11925S	1:1000
Anti-FIS1	Abcam	ab15686	1:10.000
Anti-OPA1	Cell Signaling	67589S	1:2000
Anti-GAPDH	Meridian	H86504M	1:5000
Anti-hexokinase 1	Abcam	ab154839	1:1000
Anti-hexokinase 2	Abcam	ab191838	1:1000
Anti-TOMM20	Abcam	ab186735	1:1000
Anti-α-tubulin	Sigma	T9026	1:1000
Anti-UCP2	Cell Signaling	89326S	1:1000
Anti-UCP4	Abcam	ab183886	1:1000
Anti-PKM1	Cell Signaling	7067S	1:1000
Secondary antibody	Producer	Cat. No.	Dilution
Dk-α-gt-peroxidase	Jackson ImmunoResearch	705–036-147	1:5000
Dk-α-ms-peroxidase	Jackson ImmunoResearch	715–035-151	1:5000
Dk-α-rb-peroxidase	Jackson ImmunoResearch	111–035-144	1:5000

### 2.6 Seahorse assay

Using the Seahorse XFe24 (Agilent) the oxygen consumption rate (OCR) and the extracellular acidification rate (ECAR) were measured. Based on these measurements, all other factors were calculated. Seahorse experiments were performed according to the manufacturer’s instructions. Sensors were preincubated in H_2_O at 37°C without CO_2_ overnight and changed to calibrant 1 h before the assay. Optimal cell density for each cell type was tested in prior Seahorse experiments. iAstrocytes were replated at 40.000 cells/well the day before the assay which was performed at day 45 or day 46 of differentiation. For each assay four measurements at baseline and three measurements after drug induction were performed. Each assay type was repeated three times for each cell line and included four background wells for each assay. The Seahorse DMEM medium (103575–100, Agilent) was freshly supplemented with 1 mM pyruvate and 2 mM L-glutamine and for the ATP rate and Mito Stress Test also with 10 mM glucose.

#### 2.6.1 ATP rate assay

For the ATP Rate Assay drugs were used at the final concentrations of 1.5 μM oligomycin and 0.5 μM Rotenone + Antimycin A (ROT/AA). Based on OCR and ECAR measurements, ATP production rate was calculated as follows:

**Glycolytic ATP production rate**

Glycolysis converts glucose to lactate, producing 2 ATP and 2 H^+^ per molecule of glucose. Therefore, the glycoATP production rate is equivalent to the glycolytic proton efflux rate (glycoPER):


glycoATP production rate(pmolATP/min)=glycoPER(pmolH+/min).

$${\text{mitoOCR}}^{*}\;0.5=\text{mitoPER}.$$

PER–mitoPER=glycoPER.

Where:

- 0.5 = buffer capacity of the measurement system, comprising the assay medium and XF assay conditions (instrument, sensor, labware).

**Mitochondrial ATP production rate**

The mitochondrial component of ATP production is determined from the OCR inhibited by oligomycin:

$${\mathrm{OCR}}_{\mathrm{ATP}}=\text{basal}\;\mathrm{OCR}–{\mathrm{OCR}}_{\text{Oligo}}.$$

This value is then converted to mitochondrial ATP production using the formula:

$$\text{mitoATP rate}\;(\text{pmol}\;\mathrm{ATP}/\min)={{\mathrm{OCR}}_{\mathrm{ATP}}}^{*}\;2^{*}\;P/O\;\text{ratio}.$$

Where:

- 2 ATP rate assay = number of O atoms per O₂ molecule
- P/O ratio = 2.75 (an average value validated across multiple cell types)

**Total ATP production rate**

The total ATP production rate is the sum of both pathways:


TotalATPproduction rate=glycoATP rate+mitoATP rate.

#### 2.6.2 Mito stress test

For the Mito Stress Test drugs were used at the final concentrations of 1.5 μM oligomycin, 2 μM Carbonyl cyanide-4 phenylhydrazone (FCCP) for and 0.5 μM Rotenone + Antimycin A (ROT/AA). FCCP titration was done in earlier Seahorse experiments. Based on OCR measurements, mitochondrial respiration parameters were calculated as follows:


$$\begin{array}{l} \text{Basal respiration}=(\text{Last rate measurement before first injection})\\ –(\text{Non}-\text{mitochondrial respiration rate}). \end{array}$$

$$\begin{array}{l} \text{Non}-\text{mitochondrial respiration rate}=\text{minimum rate measurement}\;\\ \;\;\;\;\;\;\;\;\;\;\;\;\;\;\;\;\;\;\;\;\;\;\;\;\;\;\;\;\;\;\;\;\;\;\;\;\;\;\;\;\;\;\;\;\;\;\;\;\;\;\;\;\;\;\;\;\;\;\;\;\;\;\;\;\;\;\;\;\;\;\;\;\;\;\;\;\;\;\;\;\;\;\;\;\text{after}\;\mathrm{ROT}/\mathrm{AA}. \end{array}$$

$$\begin{array}{l} \text{Maximal respiration}=(\begin{array}{l} \text{Maximum rate measurement}\;\\ \text{after FCCP injection} \end{array})\\ –(\text{Non}-\text{mitochondrial respiration rate}). \end{array}$$

$$\begin{array}{l} H+\text{leak}=(\begin{array}{l} \text{minimum rate measurement}\;\\ \text{after Oligomycin injection} \end{array})\\ –(\text{Non}-\text{mitochondrial respiration rate}). \end{array}$$

$$\begin{array}{l} \text{Spare respiratory capacity}\%=(\text{maximal respiration})\\ /{\left(\text{basal respiration}\right)}^{*}\;100. \end{array}$$

#### 2.6.3 Glyco stress test

For the Glyco Stress Test drugs were used at a final concentration of 10 mM glucose, 1 μM oligomycin and 50 mM 2-deoxy-glucose (2-DG). Based on ECAR measurements, glycolytic parameters were calculated as follow:


$$\begin{array}{l} \text{Glycolysis}=(\begin{array}{l} \text{Maximum rate measurement}\;\\ \text{before Oligo injection} \end{array})\\ –(\text{Last rate measurement before glucose injection}). \end{array}$$

$$\begin{array}{l} \text{Glycolytic capacity}=(\begin{array}{l} \text{Maximum rate measurement}\;\\ \text{after Oligo injection} \end{array})\\ –(\begin{array}{l} \text{Last rate measurement before}\;\\ \text{glucose injection} \end{array}). \end{array}$$

$$\text{Glycolytic reserve}\%=(\text{Glycolytic capacity})/{\left(\text{Glycolysis}\right)}^{*}\;100.$$

After Seahorse assays, cells were immediately fixated with 4% PFA and 4% sucrose in DPBS and stained with 0.4 ng/μL DAPI for 10 min at RT. DAPI was imaged with an inverted fluorescence microscope (Zeiss) with 10x magnification. The number of nuclei in each well was automatically analyzed by a macro written in Fiji and used for normalization of the Seahorse data with the Wave Software.

### 2.7 Fluorescence lifetime imaging

7–8 days before imaging, d37-39 iAstrocytes were replated onto microscope dishes (IBL, 220.110.012) at a density of 120.000 cells/dish. Cells were transduced with 1 μL AAV nanosensor 2 h after replating, once the cells had begun to attach to the plate. The lactate sensor LiLac [Koveal et al., 2022; ssAAV-2/2-shortCAG-6xHis_LiLac-WPRE-SV40p(A)] was purchased from the viral vector facility of the University of Zurich. After 24 h, virus was removed with a medium change. Cells were normally cultured until the imaging experiment at day 45/46. Before imaging, cells were cultured for at least 1 h in reference buffer. During imaging, microscope dishes were constant perfused with reference buffer containing 112 mM NaCl, 3 mM KCl, 1.25 mM CaCl_2_, 24 mM NaHCO_3_, 1.25 mM MgSO_4_, 10 mM HEPES, 2 mM glucose, 1 mM lactate, 0.1 mM pyruvate, 2 mM L-glutamine, at a rate of about 2 mL/min at a constant temperature around 34°C using a customized version of the PiFlow pump (Kassis et al., 2018). For a stable baseline, cells were given about 5–10 min to adjust to the setup. Drugs were added in addition to the reference buffer at a concentration of 5 mM sodium azide (Merck, 1.06688.0100) and 2 μM AR-C155858 (MedChem, HY-13248).

FLIM imaging was performed on a custom two-photon microscope (Mayrhofer et al., 2015) coupled to an Insight Deepsee (SpectraPhysics) femtosecond pulsed laser. A 25x water immersion objective (XLPlan N 25×/1.05w MP, WD = 2 mm, Olympus) was used for image acquisition. The excitation and emission beam paths were separated using a dichroic mirror. Emission light was further divided into specific wavelength components with dichroic mirrors at 560 and 506 nm, then focused on a PMA Hybrid 40-mod HPD detector (Picoquant) equipped with filters for CFP (475/50;) and laser rejection (770SP). Data acquisition and system control were performed using a customized version of ScanImage 3.8 and custom LabVIEW software (Version 2012; National Instruments)., while Time-Correlated Single-Photon Counting and FLIM image generation were performed using in-house instrumentation (Velasquez Moros et al., 2025). LiLac-expressing cells were excited at 870 nm and images were acquired at a 2.96 Hz with a 128×128 pixel resolution.

FLIM data analysis was conducted using FLIManalysis,^1^ a Matlab-based wrapper of the FLIMfit library (Warren et al., 2013) designed to facilitate batch processing. ROIs were selected using ImageJ and including ROI-based decay summation was performed before fitting.

### 2.8 Proteomics

For this study, we analyzed a proteomic dataset that we previously generated (de Leeuw et al., 2022) and focused on yet unpublished pathways related to energy metabolism. The detailed methodology for sample preparation, mass spectrometry acquisition and data processing has been described (de Leeuw et al., 2022). Briefly, iAstrocytes were cultured, treated and lysed in RIPA buffer before protein extraction using the iST Kit (PreOmics, Germany). Mass spectrometry analysis was performed using an Orbitrap Fusion Lumos (Thermo Scientific) coupled to an M-Class UPLC (Waters).

For the present study, we focused on proteins and pathways associated with cellular energy metabolism. The acquired raw MS data were reprocessed using MaxQuant and statistical analyses were performed in R. We conducted pathway enrichment analysis using the fgsea R/Bioconductor package, leveraging gene sets from the Molecular Signatures Database (MSigDB). Protein lists were ranked based on moderated t-statistics and enrichment scores were computed to identify significantly altered pathways. All data processing steps were conducted as previously described, with modifications specific to the analysis of metabolic pathways.

### 2.9 Metabolomics—sample preparation for LC–MS analysis of polar metabolites

iAstrocytes were cultured until day 45. Following a single PBS wash, polar metabolites were extracted using a MeOH: ACN: H2O (40:40:20) solution. The plate was manually shaken for 30 s and incubated at −80°C with closed lids for 15 min. Cells were then detached using a cell scraper, transferred to Eppendorf tubes and stored at −80°C. For each cell line three batches were collected before samples being delivered to the Functional Genomics Center Zurich. For each sample, 4 mL of extract was delivered to the Functional Genomic Center Zürich to perform LC–MS based untargeted analysis of the polar metabolites content.

The extracts have been centrifuged for 20 min at 7000 rpm and 4°C to precipitate proteins and cell debris, 3 mL of clear supernatant was progressively transferred to clean 2 mL test tubes and dried under nitrogen flow prior to solubilization of the metabolites in injection solvent (90% acetonitrile). The protein pellet was used to determine the protein content of each sample, and the protein content was later used for data normalization.

Before measuring, the samples were centrifuged for 10 min at 10.000 rpm/ 4°C and the clear supernatant was transferred to glass vials suitable for LC–MS analysis (Total Recovery Vials, Waters). In addition, method blanks, mixtures of pure standards, and pooled samples were prepared in the same way to serve as quality control for the measurements.

For heatmap visualization, log2-transformed protein expression was used. Proteins with missing values across any of the conditions were excluded. The data were then normalized by computing row-wise Z-scores (per protein). A heatmap was generated using pheatmap package in R.

### 2.10 Metabolomics—LC–MS data acquisition

Metabolites were separated on a Thermo Vanquish Horizon Binary Pump equipped with Waters Premier BEH Amide column (150 mm x 2.1 mm), applying a gradient of 10 mM ammonium bicarbonate in 5% acetonitrile pH9 (A) and 10 mM ammonium bicarbonate in 95% acetonitrile (B) from 99% B to 30% B over 12 min. The injection volume was 5 μL while the flow rate was 0.4 ul/min with column temperature of 40°C and autosampler temperature of 5°C.

The LC was coupled to Thermo Exploris 480 mass spectrometer by a HESI source. MS1 (molecular ion) and MS2 (fragment) data were acquired using negative polarization and Full MS / dd-MS^2^ (Top5) over a mass range of 70 to 1,050 m/z at MS1 and MS2 resolution of >17′500. Quality controls were run on pooled samples, reference compound mixtures, and blanks to determine technical accuracy and stability.

### 2.11 Metabolomics—untargeted metabolomics data analysis

The metabolomics dataset was evaluated in an untargeted fashion with Compound Discoverer software (Thermo Scientific). The modular data analysis workflow includes spectra selection, retention times alignment, compound detection and grouping, gap filling, background filtering and normalization (data are protein content normalized). mzCloud and mzVault have been used to score fragmentation patterns and assign MS2-based identities to the features. A filtering process was performed, leading to the manually annotated compound table, where each feature is annotated with the highest level of confidence. Filtering parameters used were the following: Signa/noise > 3, mzCloud or mzVault match >50, ppm mass error within +/− 5 ppm., match with in-house developed MS1_RT library within +/− 10 s, chromatographic peak and MS2 spectra quality.

Enrichment analysis was performed using Metaboanalyst 6.0 (Pang et al., 2024). For comparison of two groups the quantitative enrichment analysis function was selected, pathways were identified according to small molecule pathway database (SMDB) library and results were plotted using R.

### 2.12 Statistics

Normal distribution was tested by using the Shapiro–Wilk normality test and the Kolmogorov–Smirnov test. Outliers were identified with the ROUT test (Q = 1%). Differences between groups were analyzed by one-way ANOVA followed by Tukey test for multiple comparisons for normally distributed data or by the Kruskal-Wallis test followed by Dunn’s multiple comparisons test for not normally distributed data. For the Seahorse assays, 3–5 wells per cell line with each containing about 40.000 cells were analyzed per assay and each assay was repeated at least 3 times independently. One biological replicate (n) represents 1 well measured in the Seahorse assay. For FLIM, 10 cells per dish and experiment were analyzed. The 10 analyzed cells were averaged and represent 1 biological replicate (n) for our FLIM measurements. FLIM experiments were repeated at least 4 times independently. For the Western blot 1 biological sample represents 1 cell culture well, derived from an independent differentiation. For MS-based proteomics and metabolomics, 1 biological sample represents one well derived from an independent differentiation. Three biological replicates were analyzed per genotype for proteomics and metabolomics analysis.

## 3 Results

### 3.1 APOE4 decreases ATP production based on glycolysis and mitochondrial respiration

**Figure 1:**
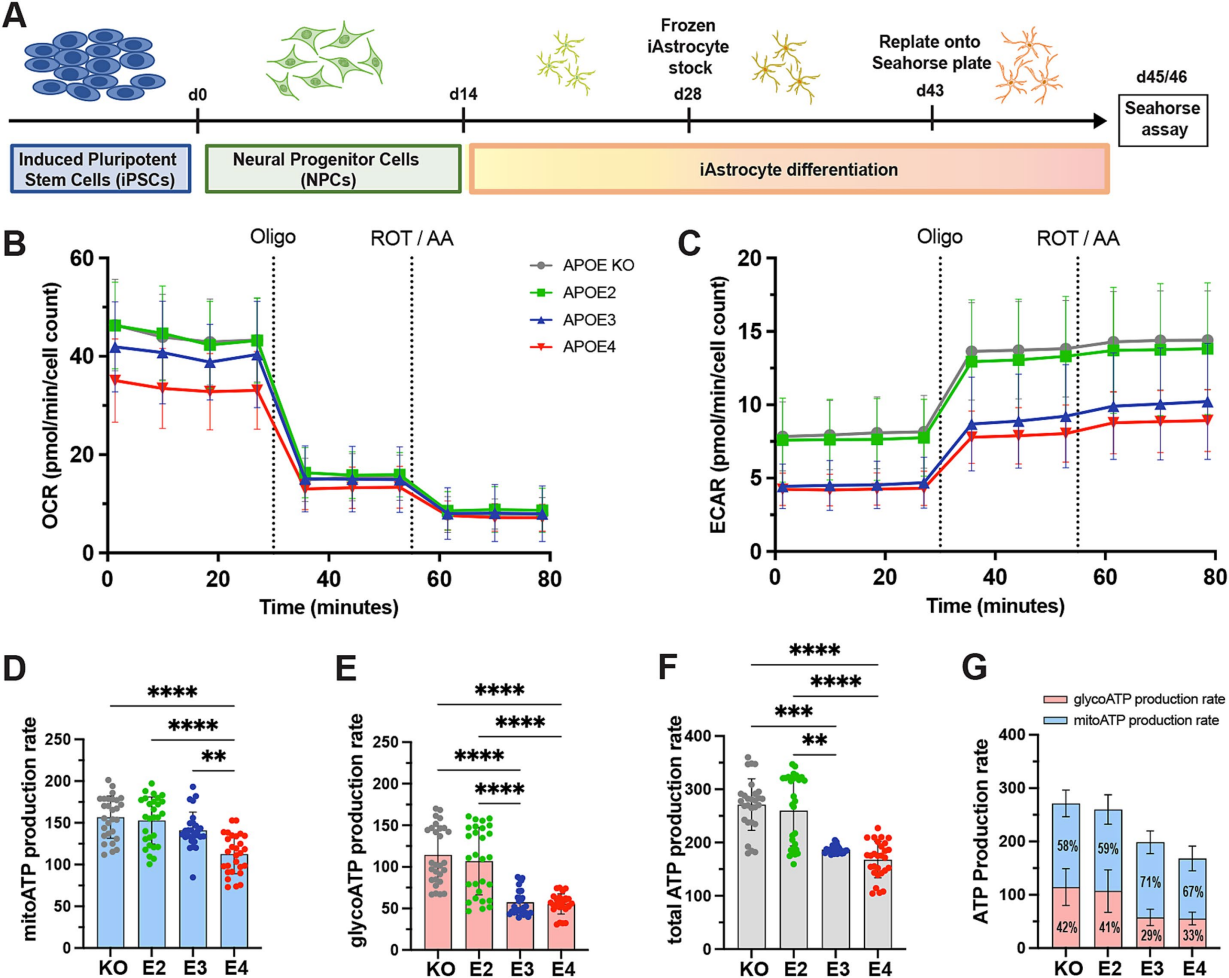
ATP production in *APOE*-isogenic astrocytes. **(A)** Schematic differentiation timeline of *APOE*-isogenic iAstrocytes for the seahorse assays. **(B)** OCR and **(C)** ECAR were measured in iAstrocytes. *APOE-KO* in gray, *APOE2* in green, *APOE3* in blue and *APOE4* in red. Oligo: Oligomycin; ROT/AA: Rotenone/Antimycin A. **(D)** Mitochondrial ATP (mitoATP) production rate, **(E)** glycolytic ATP (glycoATP) production rate and **(F, G)** total ATP production rate, calculated based on OCR and ECAR measurements. glycoATP in red and mitoATP in blue. Data was analyzed using the Kruskal-Wallis test (Dunn’s multiple comparisons test) **(D–F)**. ATP rate assay was repeated seven times independently. **p < 0.01, ***p < 0.001, ****p < 0.0001.

To determine the *APOE* effect on human astrocytes, we differentiated *APOE*-isogenic iPS cell lines (*APOE-KO*, *APOE2*, *APOE3* and *APOE4*) into functional iAstrocytes (Figure 1A). Both the *APOE*-isogenic iPSCs and iAstrocytes had already been characterized and functionally validated in previous studies (de Leeuw et al., 2022; Budny et al., 2024). To verify successful iAstrocytes differentiation, immunostaining was performed confirming the expression of astrocyte markers s100β and GJA1 (Supplementary Figure S1A). Differentiation efficiency was calculated as s100β-positive cells per total DAPI counts and reached >97% for all cell lines (Supplementary Figure S1B). To determine whether *APOE* genotypes differentially regulate the glycolytic and mitochondrial ATP production in our *APOE*-isogenic iAstrocytes, a Seahorse ATP rate assay was performed. Oxygen consumption rate (OCR) and extracellular acidification rate (ECAR) were measured four times at baseline and three times after each drug administration (Figures 1B,C). Oligomycin and Rotenone/Antimycin A (ROT/AA) were used to inhibit respiratory complex V and I/III, respectively, based on which mitochondrial and glycolytic ATP production could be analyzed. Mitochondrial ATP production was significantly lower in *APOE4* iAstrocytes than in all other *APOE* lines (E4 < E3 = E2 = KO; Figure 1D). ATP production based on glycolysis as well as total ATP production was lower in *APOE*3 and *APOE4* iAstrocytes compared to *APOE2* and *APOE-KO* (E4 = E3 < E2 = KO; Figures 1E,F). The lower total ATP production in *APOE3* compared to *APOE2* and *APOE-KO* iAstrocytes was mainly caused by a reduction in glycolysis rather than in mitochondrial respiration. Interestingly, the proportion of glycolsis-generated ATP was higher in *APOE2* and *APOE-KO* iAstrocytes (41 and 42%, respectively) than in *APOE3* (29%) and *APOE4* iAstrocytes (33%; Figure 1G).

### 3.2 APOE4 increases glycolysis

**Figure 2:**
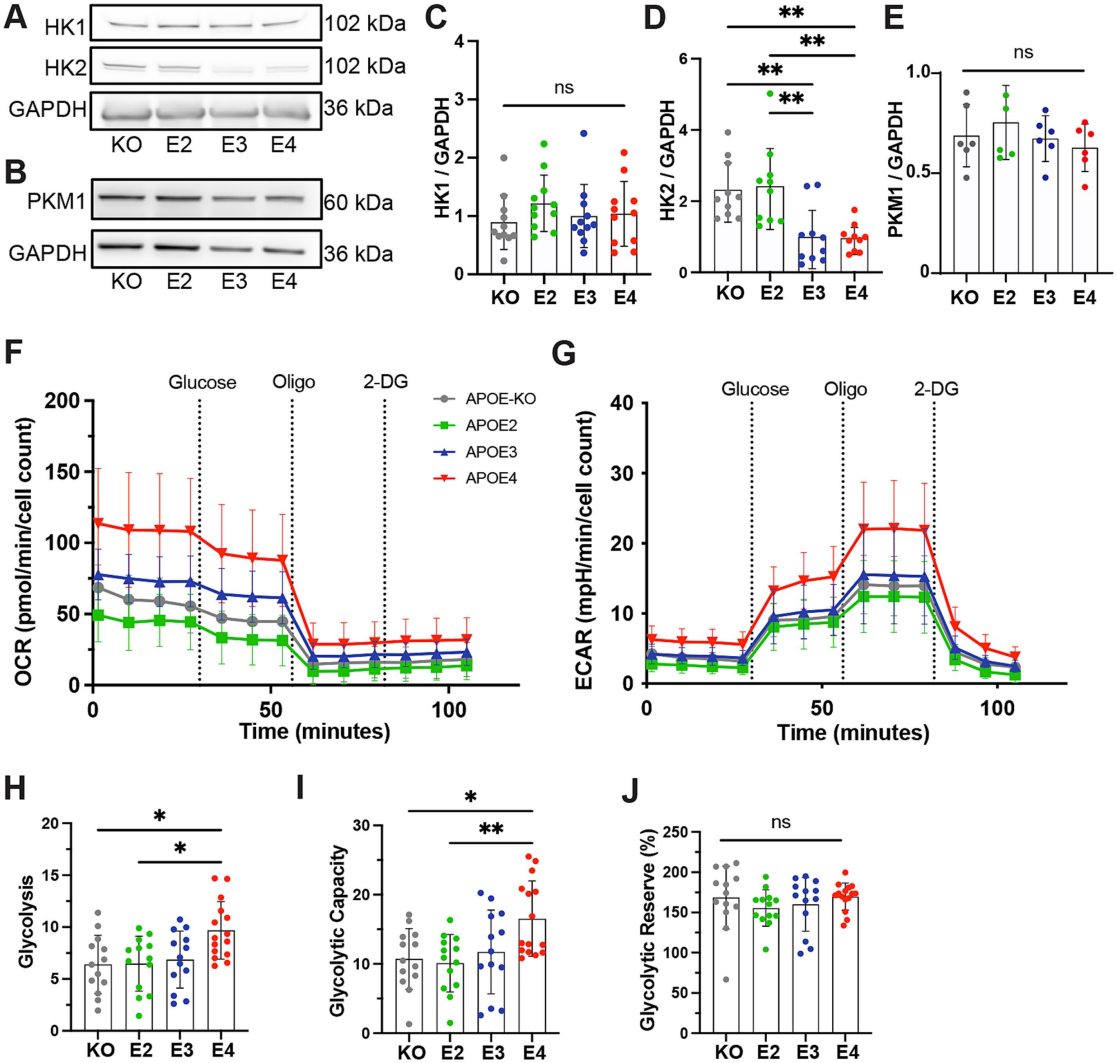
Glycolytic function in *APOE*-isogenic iAstrocytes. **(A, B)** Representative western blots of HK1, HK2, PKM1 and GAPDH in *APOE-KO*, −*E2*, −*E3* and –*E4* iAstrocytes. **(C, E)** Quantified protein levels of HK1, HK2 and PKM1 in *APOE-KO*, −*E2*, *E3* and -*E4* iAstrocytes. All protein levels were normalized to GAPDH. **(F)** OCR and **(G)** ECAR in iAstrocytes. *APOE-KO* in gray, *APOE2* in green, *APOE3* in blue and *APOE4* in red. Oligo: Oligomycin; Glu: Glucose; 2-DG: Glucose analog 2-deoxy-D-glucose. **(H)** Glycolytic function, **(I)** glycolytic capacity and **(J)** glycolytic reserve in iAstrocytes. Data was analyzed using the Kruskal-Wallis test (Dunn’s multiple comparisons test) **(D, J)** or one-way ANOVA (Tukey test for multiple comparisons) **(C, E, H, I)**. Western blots were repeated at least ten times **(C, D)** or six times **(E)** and Glyco Stress Test was repeated three times independently. ns, not significant, *p < 0.05, **p < 0.01.

We further assessed *APOE* genotype-dependent alterations of glycolytic function in iAstrocytes in more detail. Protein levels of the first enzymes in the glycolytic pathway, hexokinase 1 and 2 (HK1, HK2), were measured (Figure 2A). No differences in HK1 were observed (Figure 2C), while protein levels of HK2 were significantly lower in *APOE3* and *APOE4* compared to *APOE-KO* and *APOE2* (KO = E2 > E3 = E4; Figure 2D). This is in agreement with the reduction of glycoATP production in *APOE3* and *APOE4* (Figure 1E). Interestingly, levels of pyruvate kinase (PKM1), the enzyme that catalyzes the last step in glycolysis, was not affected by the APOE genotype (Figures 2B,E). Subsequently, the Seahorse Glyco Stress Test was performed to tease out effects glycolytic function, glycolysis as well as glycolytic capacity and reserve. OCR and ECAR were measured both at baseline and after the addition of glucose, Oligomycin and 2-deoxy-glucose (2-DG; Figures 2F,G). 2-DG is a glucose analog that inhibits glycolysis by competitively binding HK. Based on ECAR values, glycolytic properties were analyzed. *APOE4* astrocytes exhibited increased glycolysis and glycolytic capacity compared to *APOE-KO* and *APOE2* (Figures 2H,I), while glycolytic reserve remained unchanged between *APOE* lines (Figure 2J). These results indicate that *APOE4* affects glycolytic function on both protein and functional level.

### 3.3 APOE genotype does not affect lactate dynamics

**Figure 3:**
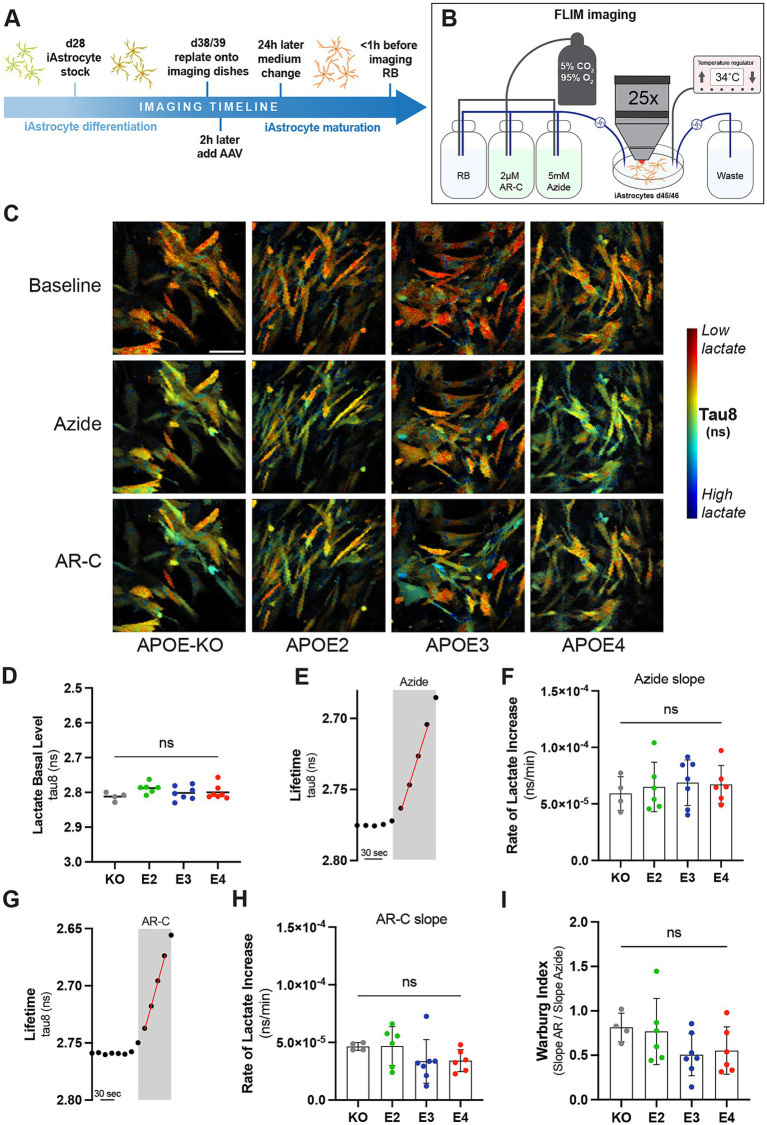
Lactate metabolism in *APOE*-isogenic iAstrocytes. **(A)** Timeline of iAstrocytes preparation for FLIM imaging with the genetically-encoded lactate sensor LiLac. **(B)** Schematic representation of FLIM setup. **(C)** Representative intensity merged FLIM images in *APOE-KO*, −*E2*, −*E3* and –*E4* isogenic iAstrocytes at baseline and with Azide or AR-C treatment. In blue low tau8 (high lactate) and in red high tau8 (low lactate). Scale bar = 100 μM **(D)** Quantification of tau8 (ns) for the basal lactate levels. **(E)** FLIM protocol to measure the lifetime change in response to 5 mM Azide. An example measurement for *APOE3* is shown. **(F)** Azide slop analysis for measuring the rate of intracellular lactate increase (ns/min). **(G)** FLIM protocol to measure the lifetime change in response to 2 μM AR-C. An example measurement for *APOE3* is shown. **(H)** AR-C slop analysis for measuring the rate of intracellular lactate increase (ns/min). **(I)** Warburg analysis based on the Azide and AR-C slop analysis. Non-parametric data was analyzed using the Kruskal-Wallis test (multiple comparisons) **(H)** and parametric data was analyzed using the ordinary one-way ANOVA (multiple comparisons) **(D, F, I)**. FLIM was repeated at least four times independently for each cell line. ns, not significant; RB, Reference buffer.

Given the observed changes in glycolysis, we further investigated glycolytic function by analyzing lactate dynamics in more detail. iAstrocytes were transduced with LiLac, a nanosensor for lactate, and fluorescence lifetime microscopy (FLIM) imaging was used to analyze intracellular lactate levels (Koveal et al., 2022; Figures 3A–C; Supplementary Video 1). Imaging was performed under cell culture-like conditions at 34°C and with carboxygenated reference buffer (RB) flow (Figure 3B). Basal lactate levels were measured during the perfusion with RB at the beginning of each experiment. No differences in basal lactate levels were observed between the lines (Figure 3D). After a constant baseline, 5 mM sodium azide was added to inhibit mitochondrial function and stimulate glycolysis. The rate of lactate increase was then assessed by slope analysis (Figure 3E; Supplementary Figure S2). Similar rates of lactate increase through azide treatment were observed between *APOE* genotypes (Figure 3F). After a constant baseline again with RB, 2 μM of the monocarboxylate transport inhibitor AR-C was added (Figure 3G). The immediate decrease in measured lifetime is equivalent to an increase in intracellular lactate and shows that iAstrocytes predominantly produce lactate. By using slope analysis, the rate of lactate increases, which represents the rate of intracellular lactate accumulation after the inhibition of lactate export through monocarboxylate transporters was determined (Figure 3H). Similar rates of lactate increase through AR-C were observed between *APOE* lines. The Warburg index, a measure of the balance between glycolytic and oxidative metabolism, was then calculated based on dividing the AR-C slope through the azide slope. This indicates whether cells rely on aerobic glycolysis rather than mitochondrial respiration for energy production. While *APOE4* and *APOE3* showed a slightly reduced Warburg index, the difference between the genotypes was not significant (Figure 3I). Taken together, APOE genotypes differentially affect mitochondrial respiration and glycolysis, but have no observable effect on lactate dynamics.

### 3.4 APOE4 affects mitochondrial respiration and increases mitochondrial stress

**Figure 4:**
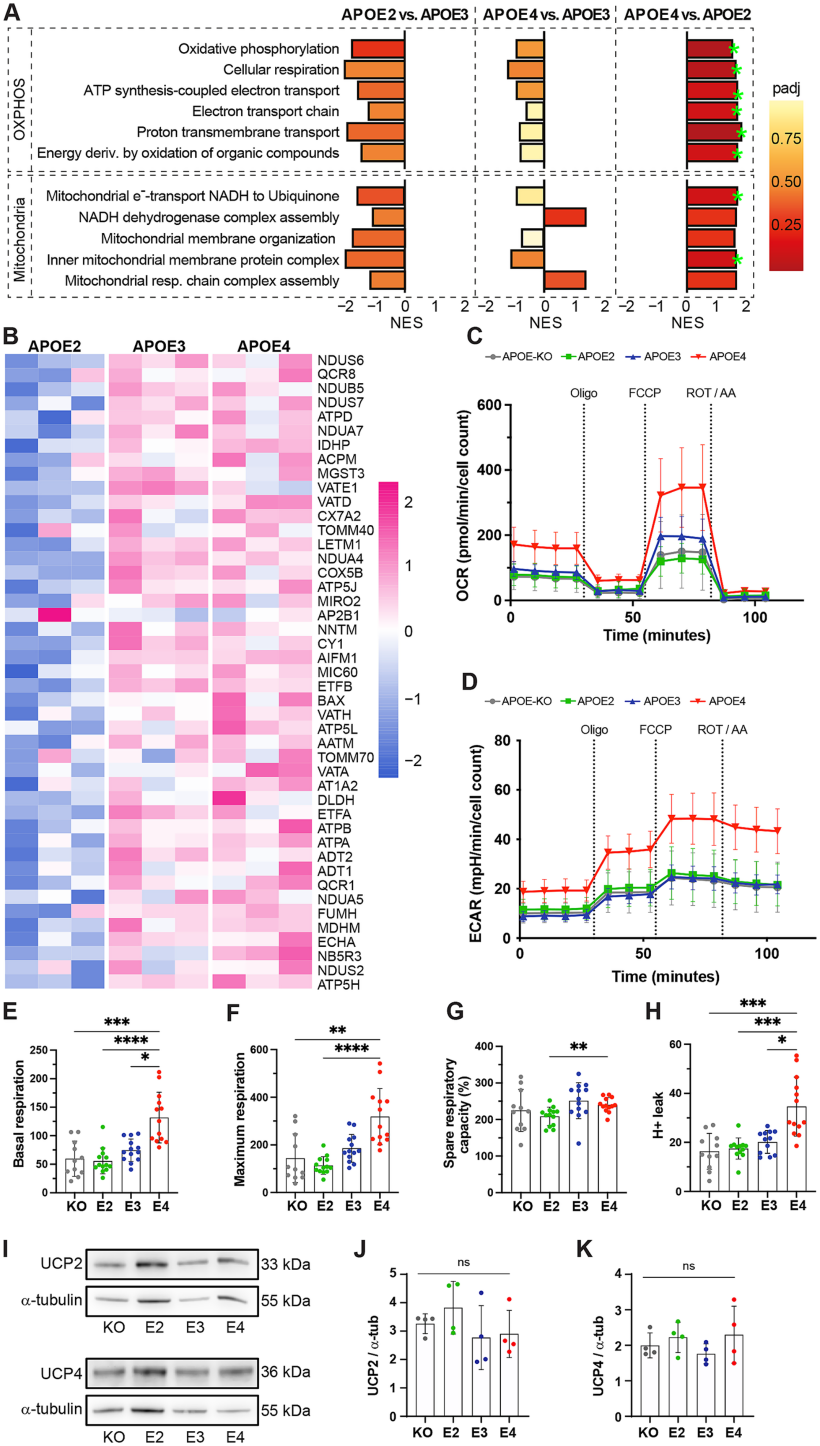
Mitochondrial function in *APOE*-isogenic iAstrocytes. **(A)** GSEA normalized enrichment scores (NESs) of proteomic lists ranked according to the t-statistic obtained for the contrast *APOE2* versus *APOE3*, *APOE4* versus *APOE3*, and *APOE4* versus *APOE2* iAstrocytes. NES is plotted on the x axis, with color-coded bars for the individual gene ontology (GO) terms. Gene sets with adjusted *p*-values <0.2 are annotated (green stars). **(B)** Heatmap of log2-transformed expression of individual leading-edge proteins. Z-scores are shown. Corresponding p-values are listed in Supplementary Table S1. **(C)** OCR and **(D)** ECAR measurements. *APOE-KO* in gray, *APOE2* in green, *APOE3* in blue and *APOE4* in red. Oligo: Oligomycin; FCCP: Carbonyl cyanide-4 (trifluoromethoxy) phenylhydrazone; ROT/AA: Rotenone/Antimycin A. **(E)** Basal respiration, **(F)** maximal respiration, **(G)** spare respiratory capacity and **(H)** proton leak in iAstrocytes. **(I)** Representative western blot images of UCP2 and UCP4. **(J, K)** Quantified protein levels of UCP2 and UCP, normalized to *α*-tubulin. Data was analyzed using the Kruskal-Wallis test (Dunn’s multiple comparisons test) **(E, F, H)** or one-way ANOVA (Tukey test for multiple comparisons) **(G, J, K)**. Western blots were repeated four times and Mito Stress Test was repeated three times independently. ns = not significant, *p < 0.05, **p < 0.01, ***p < 0.001, ****p < 0.0001.

Since *APOE4* did not only affect glycolytic function but also decreased mitoATP production, we further assessed how the different *APOE* genotypes affect oxidative energy metabolism. Therefore, we made use of data previously generated by an unlabeled mass spectrometry-based proteomic screen (de Leeuw et al., 2022). Gene set enrichment analysis (GSEA) of proteomic data showed strong upregulation of pathways involved in OXPHOS as well as mitochondrial structure and function in *APOE4* compared to *APOE2* and a tendency to downregulation in *APOE2* compared to *APOE3* (Figures 4A,B; Supplementary Table S1). It should be noted that the *APOE-KO* line had not been included in the proteomic analysis, thus proteomic data from this line was not available.

Efficient energy production relies on maintaining a healthy and functional mitochondrial network, which is regulated by dynamic reshaping events known as fusion and fission (Supplementary Figure S3A). To determine whether these processes are affected by the *APOE* genotype, proteins associated with mitochondrial fusion and fission were analyzed (Supplementary Figure S3B). No differences in the mitochondrial fusion proteins mitofusin-1/2 (MFN1/2) and optic atrophy-1 (OPA1) were detected between the *APOE*-isogenic lines (Supplementary Figure S3C–E). However, levels of mitochondrial fission marker mitochondrial fission 1 protein (FIS1) were higher in *APOE3* compared to *APOE-KO* and *APOE2* (Supplementary Figure S3F). Another mitochondrial marker, translocase of outer mitochondrial membrane 20 (TOMM20), did not show *APOE* genotype-specific differences (Supplementary Figures S3G,H).

While ATP production based on mitochondrial respiration was reduced in *APOE4* (Figure 1D), proteomic data indicated an increase in pathways related to oxidative phosphorylation (Figure 4A). To further dissect the *APOE4* effect on mitochondrial function, basal and maximal respiration as well as mitochondrial capacity and proton leak were analyzed using the Seahorse Mito Stress Test. OCR and ECAR were measured both at baseline and after the addition of Oligomycin, carbonyl cyanide-4 (trifluoromethoxy) phenylhydrazone (FCCP) and ROT/AA (Figures 4C,D). FCCP was used to induce stress, which collapses the proton gradient and disrupts the mitochondrial membrane potential. This disruption allows for unrestricted electron flow through the electron transport chain, ultimately leading to maximum oxygen consumption at complex IV. The OCR values were then used to calculate mitochondrial properties. Basal and maximal respiration was significantly higher in *APOE4* iAstrocytes compared to the genotypes (Figures 4E,F), while spare respiration capacity was increased in *APOE4* compared to *APOE2* (Figure 4G). Further, a significantly elevated proton leak was observed in *APOE4* iAstrocytes compared to all other lines (Figure 4H). To determine whether levels of mitochondrial uncoupling protein 2 and 4 (UCP2, UCP4), which are the main UCPs in astrocytes, are affected by *APOE*, western blots were performed (Figure 4I). No differences in UCP2 and UCP4 levels were found in the *APOE* isogenic lines (Figures 4J,K). This indicates that *APOE4* increases mitochondrial respiration and causes mitochondrial proton leak in the absence of changes in mitochondrial uncoupling proteins.

### 3.5 APOE genotype-dependent changes in energy metabolism-related pathways

**Figure 5:**
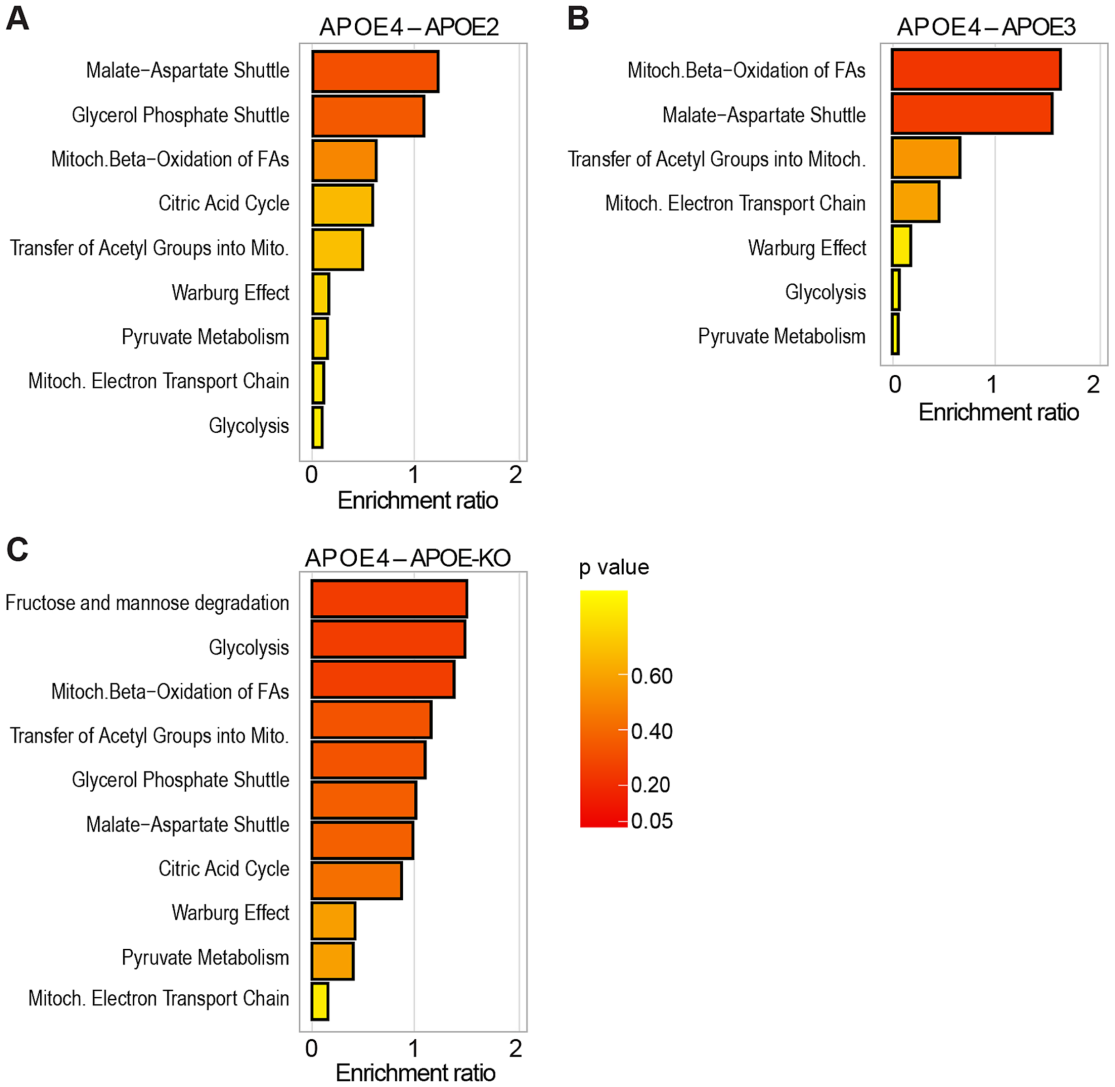
Metabolomic enrichment analysis in *APOE*-isogenic iAstrocytes. **(A–C)** Enrichment analysis of glucose and energy metabolism-related pathways, compared between the respective *APOE*-isogenic iAstrocytes and analyzed using Metaboanalyst 6.0. Pathways are ranked according to the enrichment ratio.

Next, we applied liquid chromatography–mass spectrometry (LC–MS)-based metabolomic analysis of polar metabolites followed by enrichment analysis using MetaboAnalyst 6.0 to compute differentially regulated metabolic pathways in *APOE4* iAstrocytes compared to *APOE2*, *APOE3*, and *APOE-KO* (Figure 5; Supplementary Figures S4, S5). It should be noted that the enrichment ratio does not show whether a pathway is specifically enriched in one group over the other but rather indicates that a pathway is perturbed, with some metabolites being up- and others down-regulated due to dynamic regulation of metabolic flux. We specifically looked for pathways involved in cellular energy metabolism. Nine pathways related to glucose and energy metabolism were identified when comparing *APOE4* to *APOE2* iAstrocytes (Figure 5A), while 7 pathways were identified in *APOE4* compared to *APOE3* (Figure 5B) and 11 pathways in *APOE4* compared to *APOE-KO* (Figure 5C). Some pathways were only enriched in the comparison of *APOE4* to either *APOE2*, *APOE3* or *APOE-KO*, whereas others, such as the malate–aspartate-shuttle, glycolysis, mitochondrial electron transport chain, Warburg effect or pyruvate metabolism were found in all comparisons. However, the degree of pathway enrichment varied between the lines.

When comparing *APOE4* to *APOE2*, the malate–aspartate shuttle ranked among the top 25 enriched metabolic pathways (Supplementary Figure S5A) and in the *APOE4* versus *APOE3* comparison mitochondrial beta-oxidation was among the top 25 enriched pathways (Supplementary Figure S5B). In *APOE4* compared to *APOE-KO*, fructose and mannose degradation and glycolysis were found in the top 25 pathways (Supplementary Figure S5C).

In summary, LC–MS-based metabolomic analysis indicates that *APOE4* has a major impact on human astrocyte energy metabolism, confirming our observations from proteomic analysis and functional assays.

## 4 Discussion

AD has been increasingly linked to dysregulated energy metabolism, yet the underlying mechanisms remain unclear. Using *APOE*-isogenic iPSC-derived astrocytes, we investigated *APOE*-associated differences in metabolic function. Our model shows AD-relevant proteomic pathways being specifically upregulated in *APOE4* astrocytes and downregulated in *APOE2* (Supplementary Figure S6), highlighting the biological relevance of this system.

Our data demonstrates that *APOE4* expression leads to an increase in both mitochondrial respiration and glycolysis; however, this is accompanied by inefficient ATP production. These results align partially with existing literature, which however presents conflicting findings. While some studies reported reduced glycolysis in APOE4-expressing cells (Wu et al., 2018; Fang et al., 2021; Zhang et al., 2023), others indicated a metabolic shift from oxidative phosphorylation to glycolysis (Sonntag et al., 2017; Williams et al., 2020; Farmer et al., 2021; Lee et al., 2023), with *APOE4* astrocytes exhibiting greater glycolytic activity compared to *APOE3* astrocytes. Higher oxygen consumption together with an increased glycolysis while having reduced ATP production is a sign of mitochondrial uncoupling. This occurs when the electron transport chain (ETC) continues to consume oxygen and generate a proton gradient, but ATP synthesis is less efficient because protons leak back into the mitochondrial matrix without driving ATP synthase. It leads to increased mitochondrial activity, including higher oxygen consumption as well as to increased glycolysis to compensate for ATP loss, but lower ATP production (Cadenas, 2018; Demine et al., 2019). Increased mitochondrial respiration together with decreased ATP levels can also be induced by treating cells with mitochondrial uncouplers supporting our hypothesis of *APOE4* may induce mitochondrial uncoupling (Demine et al., 2019; Shrestha et al., 2021). Recent studies have shown the role of mitochondrial uncoupling proteins (UCPs) in AD. UCP4 levels are significantly reduced in AD brain tissue and the UCP4 variant rs9472817 increases AD risk, especially in *APOE4* carriers, suggesting a connection between UCP4 and *APOE4* in disease progression (de la Monte and Wands, 2006; Montesanto et al., 2016). Overexpressing UCP4 in astrocytes in AD models prevents mitochondrial dysfunction and improves memory (Rosenberg et al., 2023). Mitochondrial dysfunction and oxidative stress, early features of AD, are linked to UCP4 downregulation due to inflammation (Thangavel et al., 2017). However, we did not observe differences in the expression of UCP2 and UCP4, two major astrocytic UCPs, in *APOE4* iAstrocytes. This suggests that mitochondrial uncoupling in *APOE4* iAstrocytes occurs independently of UCPs. Uncoupling can also be triggered by other mechanisms such as mitochondrial electron or proton leak that may be caused by mitochondrial damage (Dos Santos et al., 2024). We observed an increased proton leak specifically in *APOE4* iAstrocytes, suggesting that mitochondrial membrane alterations may contribute to the observed uncoupling. However, the exact mechanism remains to be determined and requires further investigation.

In our study, we did not observe *APOE* genotype-dependent changes in key mitochondrial fusion and fission proteins. This is consistent with our previous findings in AD patient-derived cells and *APOE*-isogenic neurons (Birnbaum et al., 2018; Budny et al., 2024). Additionally, the levels of mitochondrial proteins TOMM20, TOMM40 and TOMM70 were not significantly affected by the *APOE*, as shown by western blot (TOMM20) and proteomic analysis (TOMM40, TOMM70). This suggests that although mitochondrial function is altered by the *APOE* genotype, these changes are not driven by alterations in mitochondrial fission / fusion or by differences in mitochondrial mass. However, a more detailed investigation of mitochondrial morphology, including analysis of length and network structure, ideally via electron microscopy or imaging of mitochondrial dyes, is necessary to fully assess potential *APOE*-dependent alterations in mitochondrial structure and function. This is particularly relevant given the observed proton leak, which may reflect mitochondrial membrane damage and contribute to uncoupling as described above. We show that *APOE4* iAstrocytes exhibit lower expression of HK2 but not of HK1 or PKM1, suggesting a specific *APOE* effect on distinct enzymes rather than a general up/downregulation of glycolytic proteins. The lower HK2 levels in *APOE4* iAstrocytes may be attributed to mitochondrial dysfunctions. HK2 is typically associated with the outer mitochondrial membrane (OMM) through its interaction with the outer mitochondrial voltage-dependent anion channel (VDAC; Haloi et al., 2021). Disruption of this interaction, possibly due to changes in mitochondrial membrane potential or metabolic flux, could result in the dissociation of HK2 from the membrane, leading to its downregulation. Notably, VDAC has been considered a potential target for AD, as it plays a crucial role in mitochondrial function and VDAC malfunction can result in impaired energy production, increased oxidative stress and mitochondrial homeostasis disruption (Fang and Maldonado, 2018; Yang et al., 2024). Interestingly, HK1 expression remained unchanged in our model. This may suggest that HK1 is not as significantly impacted by mitochondrial changes in *APOE4* iAstrocytes and its expression might remain stable due to its involvement in maintaining basal glycolytic function and supporting alternative pathways like the pentose phosphate pathway (PPP), rather than rapid glycolysis for ATP production. Our findings are consistent with previous studies demonstrating significant differences in HK2 protein expression between *APOE4* and *APOE2*. However, other studies have also reported lower HK2 expression in *APOE4* compared to *APOE3*, a difference we did not observe (Wu et al., 2018; Zhang et al., 2023). This discrepancy may be attributed to the fact that HK2 expression differences between *APOE* variants become more pronounced with increasing passage numbers (Zhang et al., 2023). Similarly, Fang and colleagues found that the effects of *APOE* on OCR and ECAR in *APOE4* astrocytes were not prominent after 1 month but became evident after 2 months of culture (Fang et al., 2021). Together, these findings suggest a potential metabolic shift in *APOE4* astrocytes that intensifies over time, highlighting the importance of the time point of analysis when comparing results.

Further, *APOE-KO* and *APOE2* iAstrocytes showed higher glycoATP production and higher HK2 protein expression not only compared to *APOE4* but also to *APOE3*. A noticeable trend is observed in the glyco/mitoATP production ratio, with glycolytic ATP production being higher in *APOE-KO* and *APOE2* compared to *APOE3* and *APOE4*. This indicates that *APOE-KO* and *APOE2* cells rely more on glycolysis compared to *APOE3*. The study by Wu and colleagues showed similar findings, as differentiated N2a cells expressing APOE2 exhibited the highest HK expression and glycolytic activity. This suggests that *APOE-KO* and *APOE2* may promote a more robust glycolytic energy profile compared to *APOE3* cells (Wu et al., 2018; Zhang et al., 2023).

This study represents the first application of a genetically encoded nanosensor for lactate in *APOE*-isogenic cells, providing novel insights into intracellular lactate metabolism. Our findings reveal similar basal levels of intracellular lactate across all *APOE* variants, with no significant differences in lactate accumulation rates or Warburg index between the *APOE* variants. Notably, our iAstrocytes exhibit a Warburg index comparable to that previously reported (San Martín et al., 2013). These results suggest that mitochondrial dysfunction, as evidenced by the proton leak, may enhance oxygen consumption and overall energy expenditure. However, rather than leading to excessive lactate accumulation, glycolysis appears to be upregulated just enough to maintain metabolic balance. This implies a compensatory mechanism where pyruvate is preferentially directed toward mitochondrial oxidation rather than lactate production, thereby preserving stable intracellular lactate dynamics. Interestingly, *APOE3* cells showed reduced glycoATP but no alterations in mitoATP production, respiration or proton leak compared to *APOE-KO* and *APOE2* indicating that more research is needed to explain all *APOE* genotype dependent alterations in detail.

In contrast to many studies focusing on the comparison of only two *APOE* genotypes, we used the whole set of *APOE* isogenic lines and included *APOE-KO* in addition to *APOE2*, *APOE3* and *APOE4*. In a previous study from our lab, using the same cells as in this study, we observed differences in the expression levels of the APOE isoforms with *APOE2* iAstrocytes showing the highest and *APOE4* iAstrocytes the lowest levels of intracellular as well as secreted APOE (de Leeuw et al., 2022). However, the lower protein levels in *APOE4* iAstrocytes are not the cause for the metabolic phenotype observed in this cell line. When we compared *APOE2, APOE3* and *APOE4* iAstrocytes to *APOE-KO* cells, we observed that *APOE-KO* cells have a similar phenotype as *APOE2* but not as *APOE4*. This indicates that *APOE4* displays a gain-of-function rather than a loss-of-function effect on astrocyte metabolism. This is in agreement with our previous studies, where both *APOE-KO* iAstrocytes and iN cells showed a similar phenotype to *APOE2* but not to *APOE4*, e.g., in glutamate and Aβ uptake, cholesterol metabolism or inflammatory signaling (de Leeuw et al., 2022; Budny et al., 2024). This is also consistent with the study of Chemparathy and colleagues showing the protective function of *APOE* loss-of-variants in healthy individuals and AD patients (Chemparathy et al., 2024). Furthermore, in our metabolomic enrichment analysis, more glucose- and energy metabolism-related pathways were perturbed in *APOE4* compared to *APOE2* and *APOE-KO* (9 and 11, respectively) than compared to *APOE3* (7). This aligns with other findings, including proteomic data and ATP production, showing larger differences between *APOE4* and *APOE2/APOE-KO* than between *APOE4* and *APOE3*.

While APOE is a primarily secreted protein, it has been detected in various subcellular structures and organelles including nucleus and mitochondria (Rueter et al., 2022). Interestingly, APOE4 colocalized stronger with mitochondria than APOE3 in an *APOE* targeted-replacement astrocyte cell lines. This was accompanied by an increase in MFN1 levels in APOE4 cells (Schmukler et al., 2020). Although we did not observe *APOE* genotype-specific alterations of MFN1 or other fission and fusion proteins, we cannot exclude that differential mitochondrial or other subcellular localization of APOE2, APOE3 and APOE4 contribute to the observed effects on astrocytic energy metabolism in our study.

## 5 Conclusion

Our study provides compelling evidence that *APOE* variants differentially affect human astrocyte energy metabolism at the proteomic, metabolomic and functional level. Specifically, we demonstrate that *APOE4* drives metabolic dysregulation in iAstrocytes through a gain-of-function mechanism. Despite exhibiting increased mitochondrial respiration and glycolysis, *APOE4* iAstrocytes display reduced ATP production, likely due to mitochondrial dysfunction, as indicated by a significant proton leak. These findings enhance our understanding of AD pathophysiology and highlight potential therapeutic strategies targeting *APOE4*-driven mitochondrial dysfunction in AD.

## Data Availability

The metabolomic datasets presented in this study can be found in the following online repository: https://massive.ucsd.edu/. Dataset Identifier: MSV000097390.

## Footnotes

^1^ https://gitlab.com/einlabzurich/flimanalysis
