# Supplementary material for: APOE genotype-dependent differences in human astrocytic energy metabolism
Source: Front Cell Neurosci. 2025 Sep 1;19:1603657. doi: 10.3389/fncel.2025.1603657 (PMC12434100; doi:10.3389/fncel.2025.1603657)
Supplement: Supplementary file 1 [file Data_Sheet_1.pdf]

Suppl. figure 1

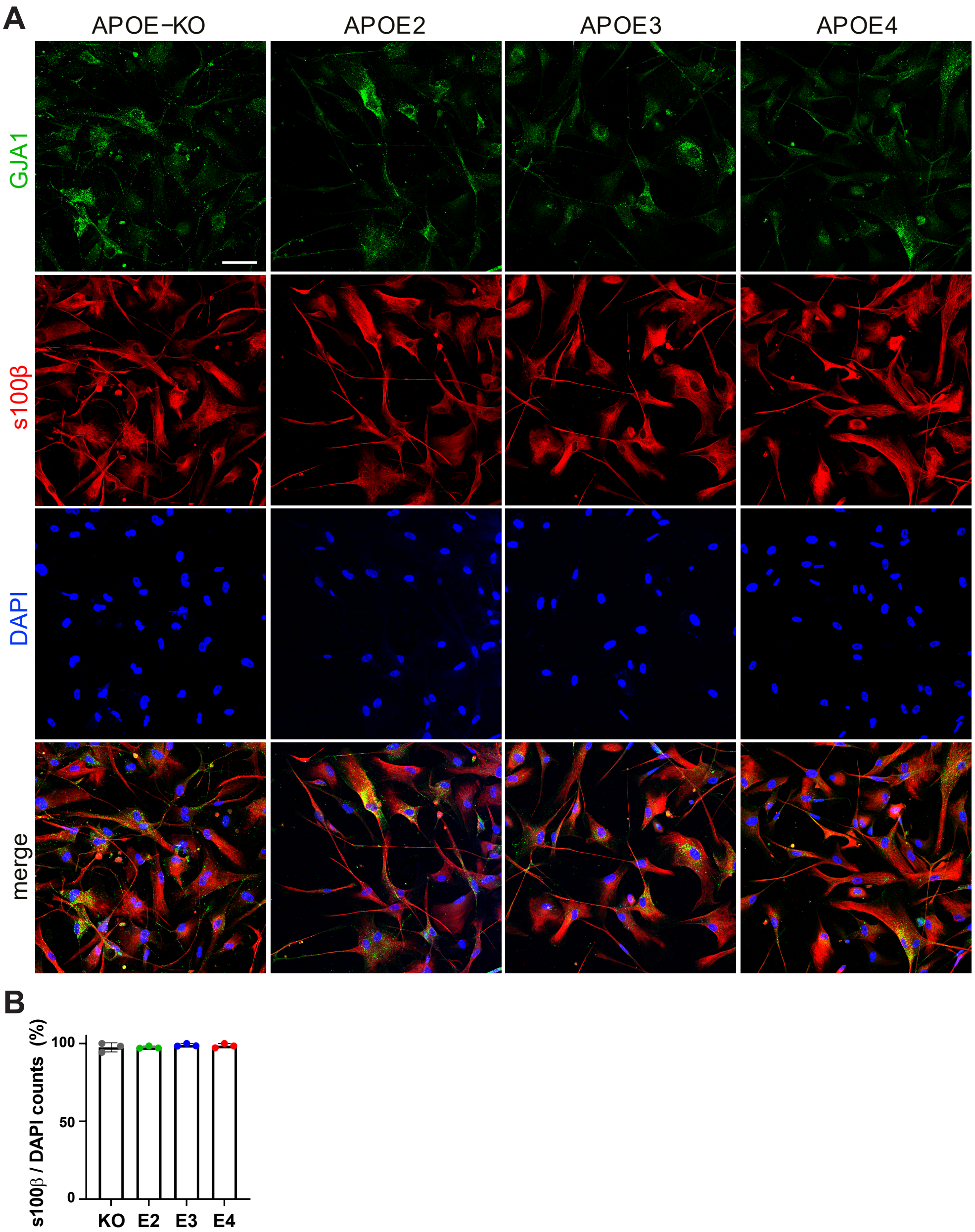

Supplementary Fig. 1: Characterization of APOE-isogenic iAstrocytes.
A: Confocal images of iAstrocytes at d44, stained for astrocyte markers s100β and GJA1. Scale bar: 100 µm
B: Differentiation efficiency plotted as s100β-positive cells per total DAPI count.

Suppl. figure 2

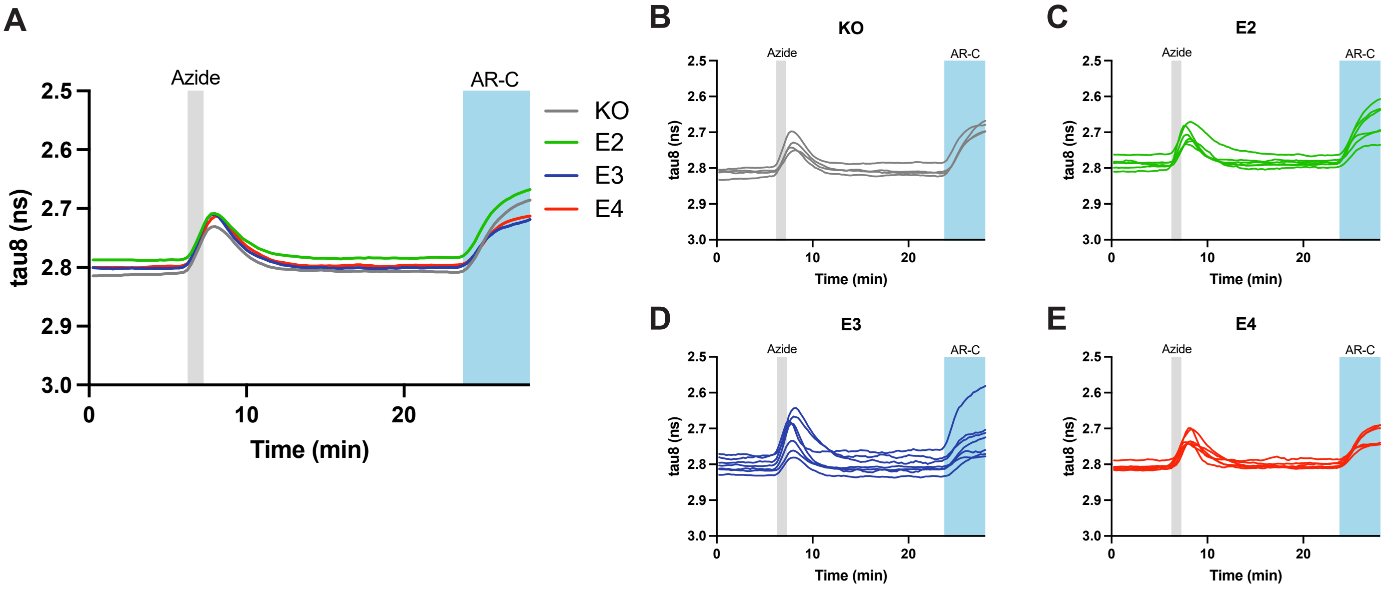

Supplementary Fig. 2: Complete FLIM measurements (tau8, ns) with the LiLac sensor over time (min). **A:** Average of all measured lifetimes for each APOE-isogenic cell line over time. **B:** Single lifetime measurements of APOE-KO iAstrocytes over time. **C:** Single lifetime measurements of APOE-E2 iAstrocytes over time. **D:** Single lifetime measurements of APOE-E3 iAstrocytes over time. **E:** Single lifetime measurements of APOE-E4 iAstrocytes over time. Grey=KO, green=E2, blue=E3, red=E4.

Suppl. figure 3

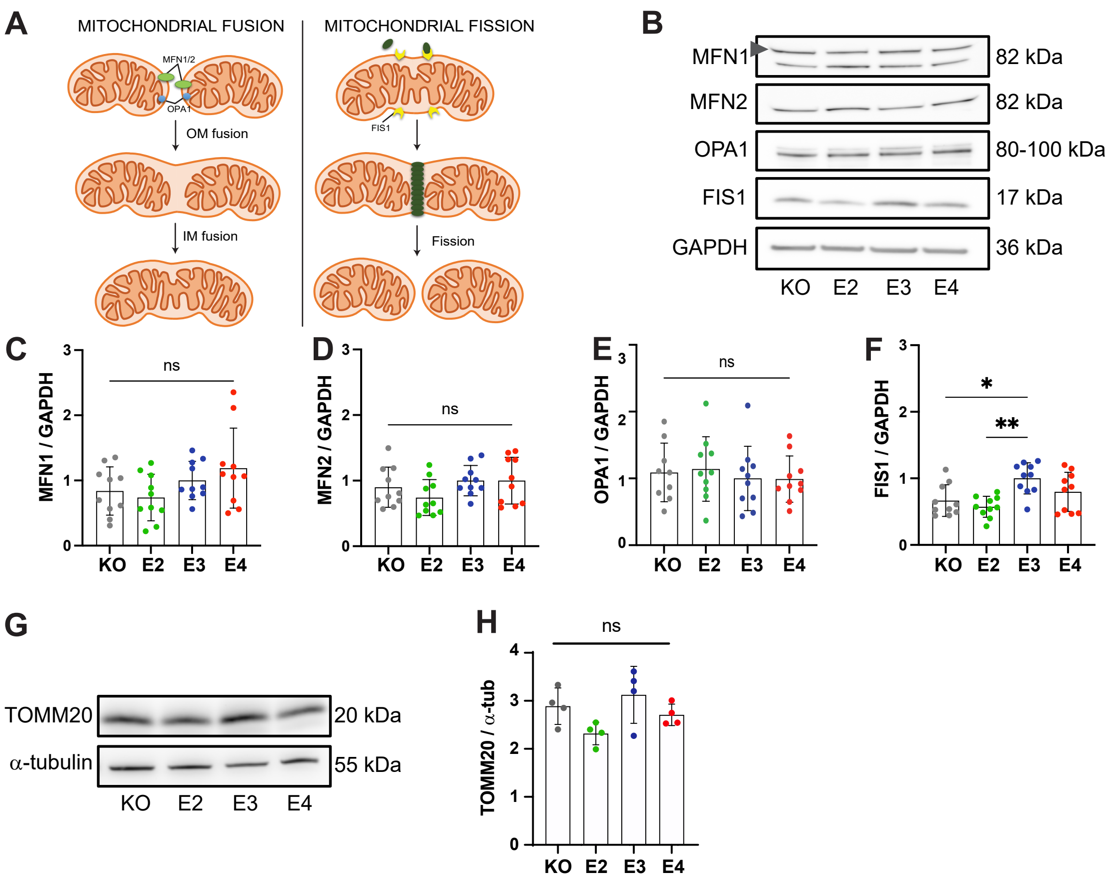

Supplementary Fig. 3: Western blots of mitochondrial proteins.
A: Schematic overview of mitochondrial fusion and fission markers. **B:** Representative western blot images of MFN1, MFN2, FIS1 and OPA1 in APOE-KO, -E2, -E3 and-E4 isogenic iAstrocytes. **C-F:** Quantified protein levels of MFN1, MFN2, FIS1 and OPA1, normalized to GAPDH. **G:** Representative western blot images of TOMM20 in APOE-KO, -E2, -E3 and-E4 isogenic iAstrocytes. **H:** Quantified protein levels of TOMM20, normalized to α-tubulin. Data was analyzed using one-way ANOVA (Tukey test for multiple comparisons). Western blots were repeated ten times (C-F) and four times (H).

Suppl. figure 4

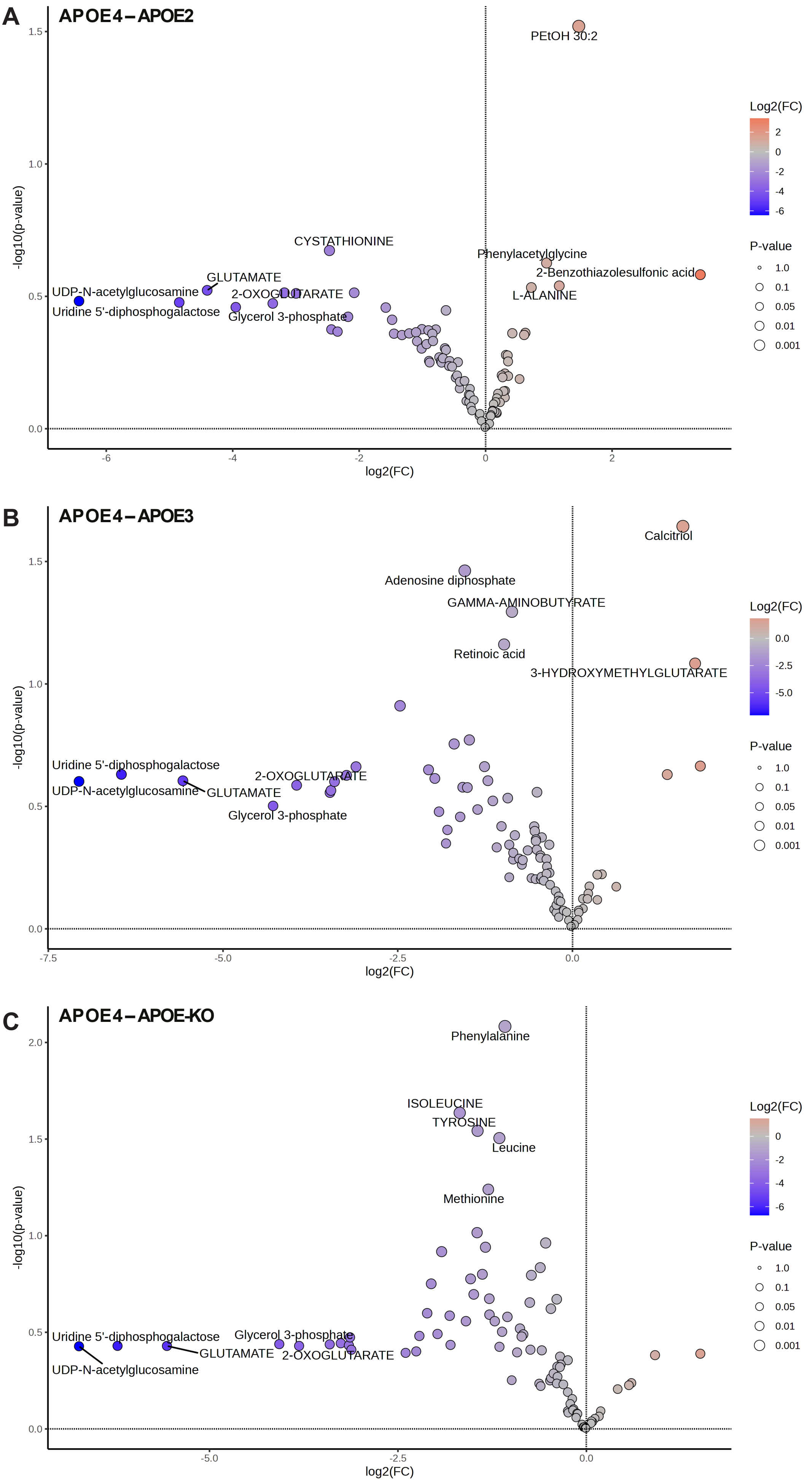

Supplementary Fig. 4: Volcano plots of metabolomic analysis, compared between the respective APOE-isogenic iAstrocytes, analyzed using Metaboanalyst 6.0. Log2 of fold change is plotted against -log10 of the p-value. Top 10 hits are annotated. Red color indicates upregulation; blue color indicates downregulation.

Suppl. figure 5

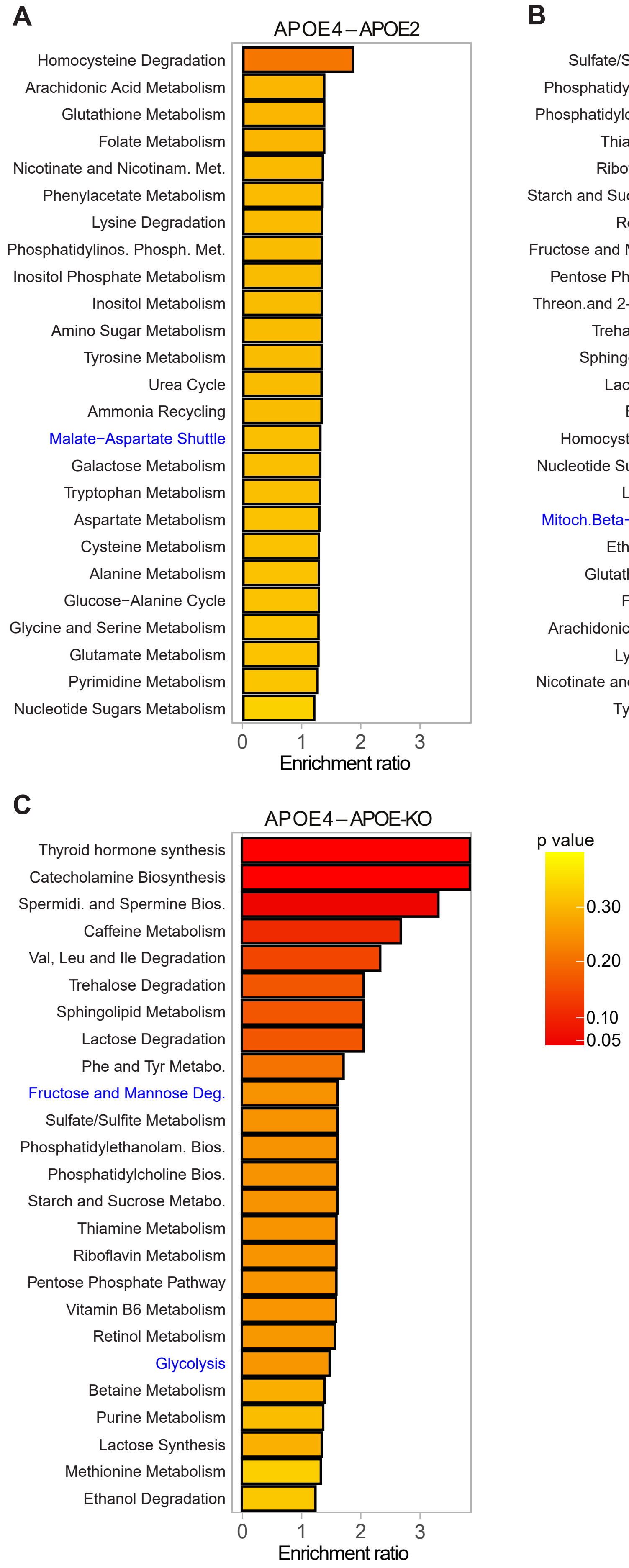

Supplementary Fig. 5: Enrichment analysis of metabolic pathways, compared between the respective APOE-isogenic iAstrocytes, analyzed using Metaboanalyst 6.0. Top 25 enriched pathways are shown. Glucose and energy metabolism-related pathwys from Fig 5A-C are highlighted in blue. Pathways are ranked according to the enrichment ratio.

Suppl. figure 6

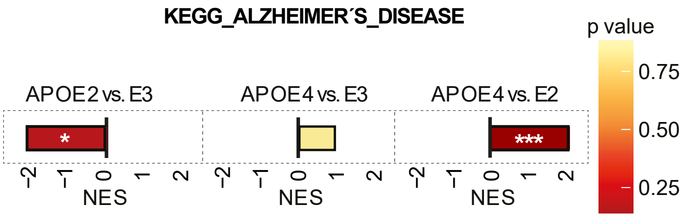

Supplementary Fig. 6: GSEA normalized enrichment scores (NES) of Alzheimer’s Disease proteomic pathway according to the KEGG database for APOE2 versus APOE3, APOE4 versus APOE3, and APOE4 versus APOE2 iAstrocytes. NES is plotted on the x axis, with color-coded bars for the individual gene ontology (GO) terms. * $p < 0.05$, *** $p < 0.001$
